# Proteome analysis of excretory-secretory proteins of *Entamoeba histolytica* HM1:IMSS via LC–ESI–MS/MS and LC–MALDI–TOF/TOF

**DOI:** 10.1186/s12014-016-9135-8

**Published:** 2016-11-22

**Authors:** Jorim Anak Ujang, Soon Hong Kwan, Mohd Nazri Ismail, Boon Huat Lim, Rahmah Noordin, Nurulhasanah Othman

**Affiliations:** 1 Institute for Research in Molecular Medicine (INFORMM), Universiti Sains Malaysia, 11800 Gelugor, Penang Malaysia; 2 Analytical Biochemistry Research Centre, Universiti Sains Malaysia, 11800 Gelugor, Penang Malaysia; 3 School of Health Sciences, Universiti Sains Malaysia, 16150 Kubang Kerian, Kelantan Malaysia

**Keywords:** *Entamoeba histolytica*, Excretory-secretory (ES) proteins, LC–ESI–MS/MS, LC–MALDI–TOF/TOF

## Abstract

**Background:**

Excretory-secretory (ES) proteins of *E. histolytica* are thought to play important roles in the host invasion, metabolism, and defence. Elucidation of the types and functions of *E. histolytica* ES proteins can further our understanding of the disease pathogenesis. Thus, the aim of this study is to use proteomics approach to better understand the complex ES proteins of the protozoa.

**Methods:**

*E. histolytica* ES proteins were prepared by culturing the trophozoites in protein-free medium. The ES proteins were identified using two mass spectrometry tools, namely, LC–ESI–MS/MS and LC–MALDI–TOF/TOF. The identified proteins were then classified according to their biological processes, molecular functions, and cellular components using the Panther classification system (PantherDB).

**Results:**

A complementary list of 219 proteins was identified; this comprised 201 proteins detected by LC–ESI–MS/MS and 107 proteins by LC–MALDI–TOF/TOF. Of the 219 proteins, 89 were identified by both mass-spectrometry systems, while 112 and 18 proteins were detected exclusively by LC–ESI–MS/MS and LC–MALDI–TOF/TOF respectively. Biological protein functional analysis using PantherDB showed that 27% of the proteins were involved in metabolic processes. Using molecular functional and cellular component analyses, 35% of the proteins were found to be involved in catalytic activity, and 21% were associated with the cell parts.

**Conclusion:**

This study showed that complementary use of LC–ESI–MS/MS and LC–MALDI–TOF/TOF has improved the identification of ES proteins. The results have increased our understanding of the types of proteins excreted/secreted by the amoeba and provided further evidence of the involvement of ES proteins in intestinal colonisation and evasion of the host immune system, as well as in encystation and excystation of the parasite.

**Electronic supplementary material:**

The online version of this article (doi:10.1186/s12014-016-9135-8) contains supplementary material, which is available to authorized users.

## Background

Amoebiasis is caused by the protozoan parasite called *Entamoeba histolytica*. The disease commonly occurs in tropical regions that lack good sanitation. Amoebiasis was ranked second to malaria as the cause of mortality due to protozoan parasites [1]. The infection often begins with the ingestion of the parasite cysts [2]. When the cysts arrive in a conductive environment, such as the intestine, they break out into the invasive trophozoite form.

Up to 90% of amoebiasis patients are mild to asymptomatic cases [3]. In symptomatic patients, amoebiasis primarily manifests as an intestinal infection, and some patients exhibit extraintestinal manifestations, mainly amoebic liver abscess (ALA). The former patients typically experience a gradual onset of abdominal cramps and pain, fever, diarrhoea, and bloody stools, while the latter condition presents with right-upper-quadrant pain, tenderness of the liver, jaundice, and nausea [4]. Diagnosis of amoebiasis includes stool microscopic examination, molecular detection, and antigen and antibody detection-based methods [5–7]. For ALA cases that do not respond to the therapy, invasive procedures such as percutaneous needle aspiration and surgery are performed [8]. Meanwhile, treatment for amoebiasis requires synergy among metronidazole, nitroimidazole, tinidazole and luminal amoebicide [9].

During infection, *E. histolytica* trophozoites release molecules called excretory-secretory (ES) proteins, which are also known as excretory-secretory antigens (ESA). ES proteins are involved in the invasion of trophozoites into the colonic mucosa by degrading the glycoside substrates and proteins of the host tissues [10–13]. Antibodies to ES proteins have been detected in the sera of both symptomatic and asymptomatic patients who have contracted amoebiasis [14].

The use of ES proteins as potential targets for diagnosis, treatment, and vaccine development for amoebiasis have been reported. The *E. histolytica* Gal/Gal-NAc lectin antigen is being utilized in commercial antigen detection tests such as the TechLab *E. histolytica* II ELISA (TechLab Inc). A study on ES proteins showed the diagnostic potential of pyruvate phosphate dikinase (PPDK), and its recombinant form has been used to develop a lateral flow dipstick test [15, 16]. In terms of treatment, auronofin has been identified as an effective drug which targets thioredoxin reductase, an ES protein of *E. histolytica* [17]. Furthermore, Gal/Gal-NAc lectin also showed potential as a vaccine candidate against *E. histolytica* [18].

A study on the ES proteins of *Trypanosome* sp. using proteomic tool has uncovered a range of proteins which include unfolding and degradation classes of proteins, such as serine, cysteine proteases, and metallopeptidases. These proteases play a part in the physiological and pathological functions that favour invasion of the parasite, its growth in hostile host conditions, evasion of the host immune defence, and hydrolysis of host proteins [19].

The main aim of the present study was to better understand the complex ES proteins of *E. histolytica.* It involved liquid chromatography-mass spectrometry analysis, in which two types of ionisation techniques, namely, electro spray ionization (ESI) and matrix assisted laser desorption ionisation (MALDI), were used in order to obtain the maximum number of protein hits. The combination of both techniques has allowed us to provide improved proteome coverage of the ES proteins.

## Methods

### Production of *E. histolytica* ES proteins

*Entamoeba histolytica* HM1:IMSS trophozoites were axenically cultured in TYI-S-33 medium supplemented with 12.5% bovine serum (GIBCO, New Zealand) and 1× Diamond’s vitamin Tween 80 (Sigma-Aldrich), pH 6.8 at 36 °C. The culture medium was changed every 48 h. The trophozoites were harvested and rinsed three times with protein-free RPMI medium 1640 (ref no.:31800-022) supplemented with 0.1% l-cysteine and 0.02% ascorbic acid (RPMI-C-A medium), by centrifuging at 440×*g* for 2 min at room temperature (RT). Trophozoites at a density of 0.5 × 10^6^ cells per ml were then seeded into culture tubes containing 80% (8 mL/tube) RPMI-C-A medium and incubated at 36 °C for 6 h. Subsequently, the culture tubes were chilled on ice for 5 min and then centrifuged at 22×*g* at 4 °C. To protect ES proteins from proteolytic activity, iodoacetamide (IAA) was added to the resultant supernatant at a final concentration of 1 mM. The supernatant was then pooled and centrifuged at 10,000×*g* for 5 min at 4 °C and filtered through 0.2 µm filter (Sartorius Stedim, Germany). Subsequently, the supernatant was concentrated 1000 times using a spin filter with 5 kDa molecular-weight cut off (Vivapsin, Sartorius), and a cocktail of protease inhibitors (Roche, Germany) was added [20]. Protein samples and RPMI-CA medium (concentrated 100X, as control) were reduced with 0.284 M β-mercaptoethanol by boiling for 5 min. Subsequently the reduced protein was electrophoresed in 10% polyacrylamide resolving gel, pH 8.8 containing 0.4675 M Tris base, 0.1% SDS, 60 µL of 10% ammonium persulphate and 6 µL of TEMED. Before staining, resolving gel was rinsed with dH_2_O three times (5 min/wash) with gentle agitation. The gel was then stained with RAMA stain which comprised 0.05% Coomasie Briliant Blue (CBB) R250, 10% acetic acid, 15% methanol and 3% ammonium sulphate, for at least 30 min with gentle agitation. The stained gels were then destained with dH_2_O until the protein bands were clearly seen.

### In-solution digestion of ES proteins

One hundred µg of ES proteins were solubilised with 0.05% RapiGest SF surfactant (Waters, USA) at 80 °C for 15 min. The sample was then reduced with 5 µL of 100 mM dithiothreitol (DTT) and incubated at 60 °C for 15 min. The sample was then cooled to RT before alkylating it using 5 µL of 20 mM IAA in the dark at RT for 30 min. Two µg of trypsin (Promega) was then added to the sample and incubated at 37 °C for 16 h. Subsequently, the digestion was stopped by adding trifluoroacetic acid (TFA) at a final concentration of ~1% and incubating at 37 °C for 20 min. The digested proteins were then centrifuged at 14,462×*g* for 15 min. The supernatant was collected and filtered through a 0.45 µm filter (Sartorius Stedim, Germany) before proceeding to peptide separation by liquid chromatography.

### Liquid chromatography and MALDI mass spectrometry

The separation of peptides prior to MALDI–TOF/TOF was performed using eksigent nanoLC ultra 1D plus (Eksigent,Germany) linked to an automated spotter (Eksigent, Germany). To achieve spatial discrimination of the peptide mixtures, 2 µL of the digested proteins was auto-loaded and packed into a C18 column. Mobile phase buffer A consisted of 0.1% TFA in 2% Acetonitrile (ACN) and 97.9% water while mobile phase buffer B consisted of 0.1% TFA in 98% ACN and 1.9% water. The gradient pump was set to elute the peptides with 20–80% acetonitrile for a duration of 165 min and at a flow rate of 0.3 µL/min. The eluted peptides were then automatically spotted between the 30th and 160th min of the gradient phase, with a 5 mg/ml α-cyano-4-hydroxycinnamic acid (CHCA) matrix flow of 1.8 µL/min for a duration of 25 s for each spot. MALDI–MS and MS/MS were performed in an automated LC mode on the AB Sciex TOF/TOF™ 5800 system (AB Sciex, USA). Mass spectra from each spot were obtained in the *m/z* range from 800 to 4000, whereby up to 500 laser shots were accumulated per spectrum. The signal-to-noise (S/N) ratio was set to a minimum of 10, and the spots with the highest intensity of precursor ion were subjected to MS/MS analysis. A maximum of ten precursors were allowed for the MS/MS analysis; for each spectrum, up to 2000 laser shots were accumulated per spectrum, and the S/N were set to a minimum ratio of 15 S/N. The mass spectrometry data were analysed using ProteinPilot™ Software 4.5 and searched using Paragon against a combined AmoebaDB_4.1 and cRAP (‘protein contaminants database’) which was set to search with the following parameters: false discovery rate of <1%, detected protein threshold of >0.47 (66%), and competitor error margin of 2.00. The cRAP includes the possible contaminant proteins in this study such as BSA and keratin (http://www.thegpm.org/crap/).

### Liquid chromatography and ESI mass spectrometry

The analyses were performed using an LTQ-Orbitrap Velos Pro (Thermo Scientific, San Jose, CA, USA) mass spectrometer coupled with the Easy-nLC II (Thermo Scientific, San Jose, CA, USA) nano liquid chromatography system. The chromatographic separation of tryptic-digested peptides was performed using Easy-Column C18-A2 (100 × 0.75 mm i.d., 3 µm; Thermo Scientific, San Jose, CA, USA) coupled with pre-column (Easy-Column, 20 × 0.1 mm i.d., 5 µm; Thermo Scientific, San Jose, CA, USA) at a flow rate of 0.3 µL/min and sample injection volume of 10 µL. The pre-column was equilibrated for 15 µL at a flow rate of 3 µL/min whereas the analytical column was equilibrated for 4 µL at a flow rate of 0.3 µL/min. The running buffers used were (A) deionised distilled water with 0.1% formic acid and (B) acetonitrile with 0.1% formic acid. The samples were eluted using a gradient of B from 5 to 100% in 100 min. The fragmentation technique used was collision induced dissociation (CID). De Novo sequencing and database matching against a combined AmoebaDB_4.1 and cRAP (‘protein contaminants database’) was performed using Peaks Studio Version 7 (Bioinformatics Solution, USA). The cRAP includes the possible contaminant proteins in this study such as BSA and keratin (http://www.thegpm.org/crap/). The parameters used in De Novo sequencing were precursor mass tolerance at 0.1 Da and fragment mass error tolerance at 0.8 Da. Subsequently for the database matching, the parameters were carbamidomethylation and methionine oxidation as fixed modifications, 2 maximum missed cleavages, false detection rate (FDR) <0.1% and parent mass and 0.1 Da precursor mass tolerance. Besides, significant score (−10lgP) for protein acceptance were set at >20, whereas minimum unique peptide was set at 1.

### Functional group analysis

The list of proteins obtained from the combination of both analyses was then assigned functional categories using the Panther Classification System (pantherdb.org).

## Results and discussion

### Protein identification

**Fig. 1:**
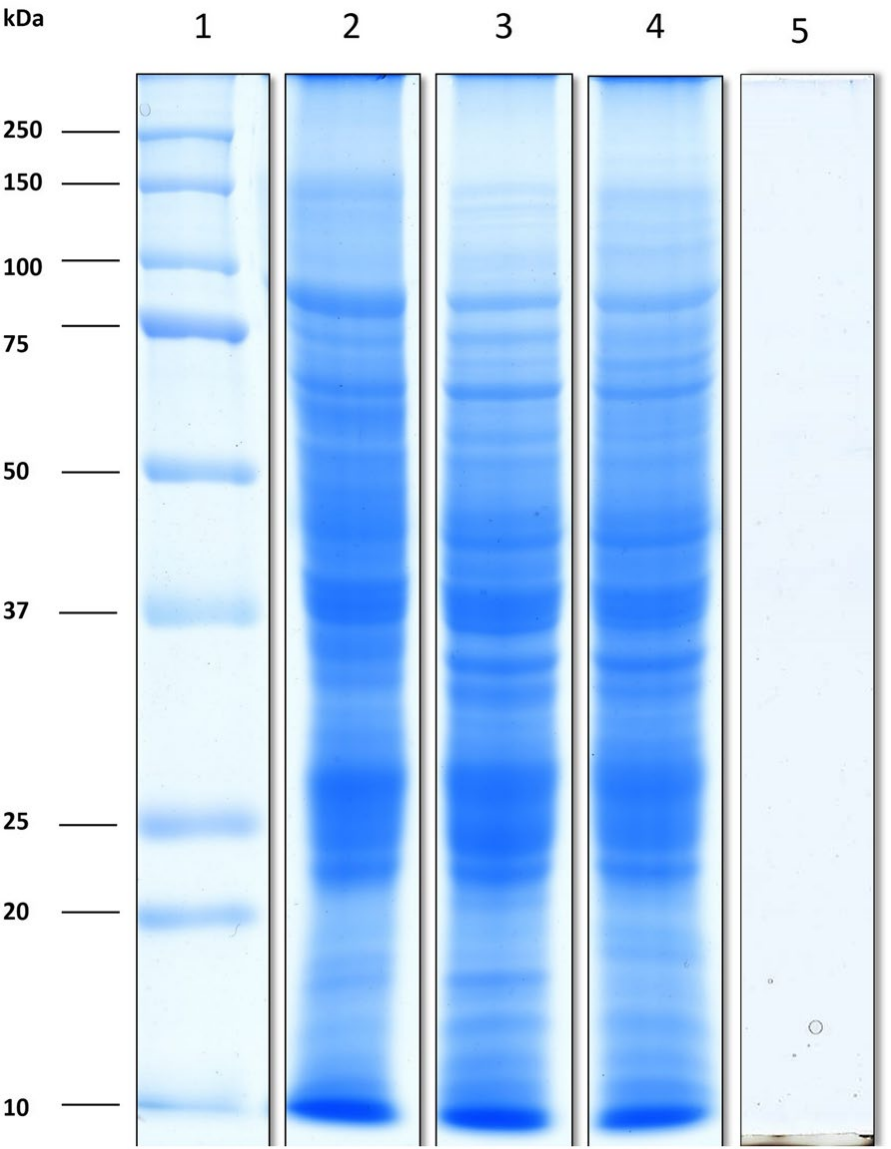
SDS-PAGE profile of excretory-secretory (ES) proteins of *E. histolytica.* ES proteins from *E. histolytica* separated by 10% SDS-PAGE. *Lane 1* protein ladder (BioRad, USA); *lane 2, 3*, and *4*, ES proteins from three independent replicates; *lane 5*, concentrated RPMI-C-A as control

In this study, ES proteins and concentrated RPMI-CA medium were resolved in 10% SDS-PAGE, and stained with RAMA solution. Visual observation of distinct protein bands indicated good protein quality while the concentrated protein free RPMI-CA medium (control) showed no protein band (Fig. 1). Furthermore, no protein smearing was observed, and this also suggested that minimal degradation of the proteins had occurred. Thus, the ES proteins were suitable for downstream mass spectrometry analysis.

**Fig. 2:**
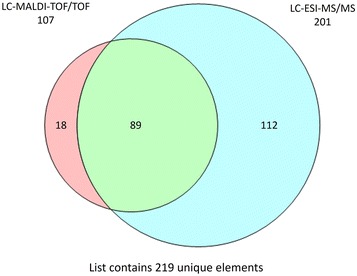
Excretory-secretory (ES) proteins detected by LC–ESI MS/MS and LC–MALDI TOF/TOF. Venn diagram depicting 219 ES proteins detected through the complementary use of LC–ESI MS/MS and LC–MALDI TOF/TOF. The list of proteins found in LC–ESI MS/MS and LC–MALDI TOF/TOF can be found in Additional files 1 and 2

We have identified 219 proteins that were excreted-secreted into the extracellular environment, with 18 proteins unique to MALDI and 112 proteins unique to ESI, while 89 proteins were identified by both systems (Fig. 2). Two replicates were analysed with ESI, and three replicates with MALDI TOF–TOF. The proteins there were considered to be significant fell into one of the following categories: a protein that was detected in both replicates using LC–ESI–MS/MS; a protein that was detected in at least two replicates using LC–MALDI–TOF/TOF; and a protein that was detected in only one replicate but detected by both mass-spectrometry systems. The details of the analyses are given in the Additional files 1 and 2. Tables 1 and 2 show representatives of the ten highest protein scores from ESI and MALDI respectively. The results of all protein identifications, peptides summaries, De novo and contaminant proteins analyses are provided in the Additional files 1, 2, 3, 4 and 5.

**Table 1:** Top 10 proteins of highest scores detected by LC–ESI–MS/MS

No	Accession	Name	−10lgP	Matched peptides	Sequence coverage (%)
*Top ten proteins identified through LC–ESI MS/MS*					
1	EHI_150490	Aldehyde-alcohol dehydrogenase 2 putative	149.46	56	73
2	EHI_051060	Pyruvate:ferredoxin oxidoreductase	148.56	50	53
3	EHI_009530	Pyruvate phosphate dikinase	146.95	47	60
4	EHI_006980	Gal/GalNAc lectin subunit Igl1	135.22	35	39
5	EHI_077500	Galactose-specific adhesin 170kD subunit	134.04	36	34
6	EHI_030750	Hypothetical protein	133.28	39	50
7	EHI_133900	Galactose-inhibitable lectin 170 kDa subunit putative	133.14	37	35
8	EHI_044970	Malic enzyme putative	132.55	33	73
9	EHI_042370	Galactose-specific adhesin 170kD subunit putative	130.92	32	31
10	EHI_166920	Hypothetical protein	130.71	35	43

−*10lgP* A representation of *p* value, whereby the higher the −10lgP value is, the more significant the match is. A threshold score of above 20 signifies relatively high confidence

*Matched peptides* The number of high-confidence supporting peptide

*Sequence coverage* The percentage of protein sequence covered by peptide sequence

**Table 2:** Top ten proteins of highest scores detected by LC–MALDI–TOF/TOF

Accession	Description	Score (%)	Matched peptides	Sequence coverage (%)
*Top ten proteins identified through LC–MALDI TOF/TOF*				
EHI_160940	Aldehyde-alcohol dehydrogenase 2, putative	28.67	17	31.8
EHI_150490	Aldehyde-alcohol dehydrogenase 2	28.67	17	31.8
EHI_130700	Enolase, putative	25.52	13	53.2
EHI_009530	Pyruvate phosphate dikinase	20.16	8	30.9
EHI_044970	Malic enzyme, putative	11.96	5	30.4
EHI_178960	Acetyl-CoA synthetase, putative	10.34	5	20.6
EHI_125950	Alcohol dehydrogenase, putative	10	5	23.2
EHI_050330	Malic enzyme, putative	9.84	4	26.7
EHI_051060	Pyruvate: ferredoxin oxidoreductase	8.67	3	16.5
EHI_107290	Actin, putative	8.44	5	8.44

*Score (%)* Total (ProtScore), a measure of the total amount of evidence for a detected protein. The Total ProtScore is calculated using all the peptides detected for the protein

*Matched peptides* The number of distinct peptides having at least 95% confidence

*Sequence coverage* The percentage of matching amino acids from identified peptides having confidence greater than 0 divided by the total number of amino acids in the sequence

The protein-free media (RPMI-C-A) used in this experiment has previously been shown to support 95% viability of *E. histolytica* trophozoites for up to 8 h [21]. Nevertheless, there may still be a small number of dead *E. histolytica* trophozoites that were lysed and released proteins into the medium. Therefore it was possible that among the 219 proteins identified, a small proportion may be non-ES proteins of the amoeba.

The results of this study highlighted the important contributions of each mass-spectrometry system and showed that the use of only a single method does not provide good protein identification of a complex sample. This observation is consistent with the report by Bodnar [22] in which they observed that 16 and 22% proteins were identified uniquely by ESI and MALDI respectively.

This study showed that the ESI system identified more ES proteins compared to MALDI. The nature of the peptides and the ionization processes may have contributed to this unequal results [23, 24]. As described by Stapels and Barofsky [25], the ESI system tends to ionize hydrophobic peptides while MALDI tends to identify basic and aromatic peptides. MALDI is able to detect higher proportions of arginine (R) terminating peptides while ESI favours lysine (K) terminating peptides [26]. In addition, compared to MALDI, ESI tends to detect lower mass peptides [27, 28].

We have identified 25% (52) well-annotated proteins, as well as 65% (136) putative, and 10% (21) hypothetical proteins. Pawlowski [29] discussed the importance of presenting uncharacterized proteins, as these may hold novel and promising discoveries. Therefore, we have included putative and hypothetical proteins, as they embody unexplored information and clues for new source of biological markers.

### Functional protein classification

**Fig. 3:**
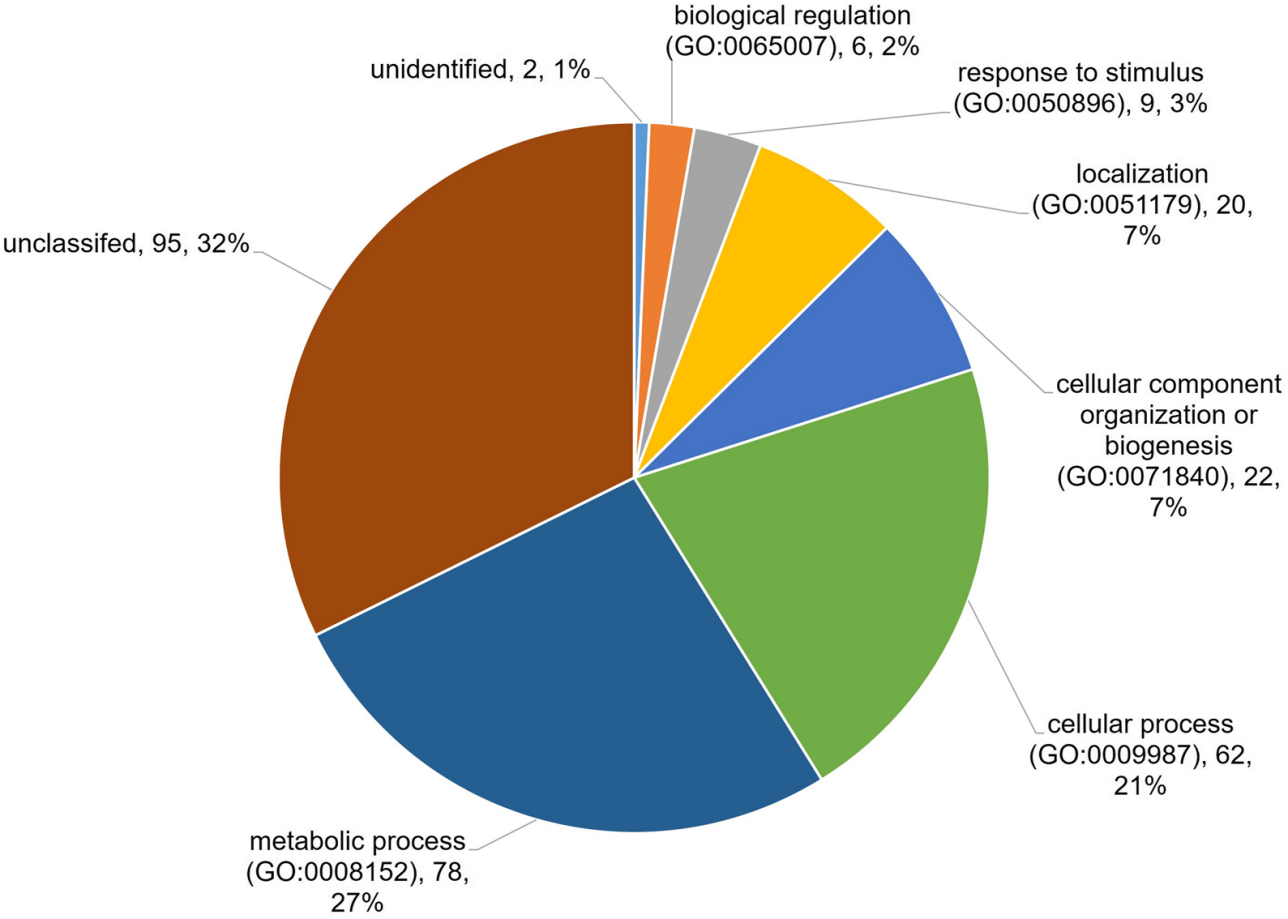
Biological function. Categories of ES proteins grouped according to the biological functions. The classifications were generated using Panther version 11.0 released 2016-07-15

**Fig. 4:**
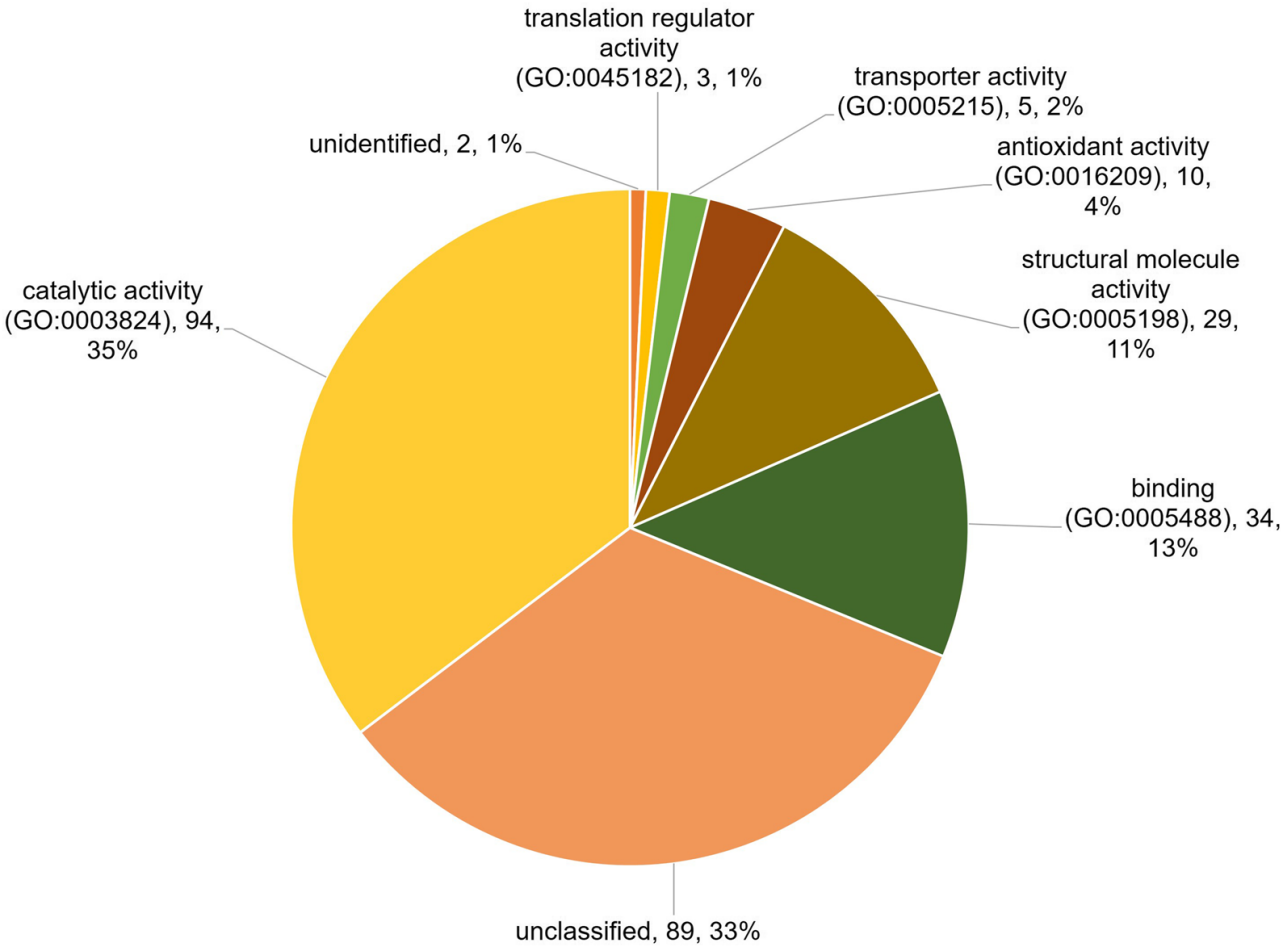
Molecular function. Classification of ES proteins into molecular function categories. The classifications were generated using Panther version 11.0 released 2016-07-15

When categorised according to biological functions (Fig. 3), a total of 78 (27%) proteins were associated with the metabolic process, 62 (21%) were involved with the cellular process, 6 (2%) were biological regulation proteins, 20 (7%) were proteins involved in localization, 22 (7%) were cellular component proteins, and 9 (3%) proteins were associated with response to stimulus. The largest category by molecular function (Fig. 4) was associated with catalytic activity whereby there was a total of 95 (35%) proteins, followed by 34 (13%) binding proteins, 29 (10%) proteins involved in structural molecule activity, 10 (4%) proteins involved in antioxidant activity, 5 (2%) proteins with transporter activity, and 3 (1%) translation regulator proteins.

The top two categories in the biological and molecular functions were metabolic process and catalytic activity. The major protein representatives in these two categories were peroxiredoxins (PRXs) (EHI_139570, EHI_145840, EHI_121620, EHI_122310, EHI_123390, EHI_061980, EHI_001420, EHI_201250, and EHI_114010). PRXs are involved in redox metabolism, oxidoreductase activity, and peroxidase activity. The presence of these antioxidant enzymes in the extracellular environment aids in the defence mechanism of the parasite against reactive oxygen species (ROS) that is imposed by the host immune system [30]. Wassmann et al. [31] reported an increased expression of PRXs in metronidazole resistant *E. histolytica*. They also showed that there is an increased expression of iron-containing superoxide dismutase (SOD), and hypothesised that PRXs and SOD play a role in the resistance of metronidazole in *E. histolytica*. In the present study, we have also identified superoxide dismutase [Fe];SODB (EHI_159160), which is involved in the metabolic process.

In another finding, PRX showed interaction with *N*-acetyl-galactosamine inhibitable (GalNAc) lectin in protecting the parasite [32]. It is also believed that the recruitment of PRX by lectin leads to a signal transduction that protects the parasite from oxidative stress [33]. The Gal/GalNAc lectin is an adhesin involved in the attachment of the parasite to the host. This was observed when the parasite failed to engage adhesion and contact-dependant cytotoxicity with cells that lacked terminal Gal/GalNAc residues [34, 35]. We have identified twelve GalNAc lectin-related proteins, i.e., EHI_006980, EHI_065330, EHI_148790, EHI_035690, EHI_058330, EHI_049690, EHI_042370, EHI_133900, EHI_012270 and EHI_077500, although this group of proteins does not fall into any of the classified functional annotations by PantherDB. Nonetheless, GalNAc lectin proteins are well studied, and their potential has already been explored and exploited. They contribute to the virulence of the parasite by their adherence to the host tissue, and cause contact-dependent cytolysis of target cells; they are also involved in phagocytosis and contribute to resistance to cell lysis inflicted by the host [36]. The GalNAc lectin protein was also reported by Wong et al. [15] as one of the components of excretory-secretory antigens of *E. histolytica*. In addition, the abundance of the Gal/GalNAc lectin protein on the surface of the parasite and its antigenic property have allowed it to be used for antigen detection in the TechLab *Entamoeba histolytica* II ELISA kit (TechLAB Inc.) [37].

Another family/sub family group of proteins that was included in the metabolic process is 60 s acidic ribosomal proteins (EHI_138770, EHI_052610). To the best of our knowledge, these are putative proteins with only two other reports on *E. histolytica* [38, 39]. They are strongly acidic proteins that are commonly found on the surface of the ribosome in all organisms [40]. Eukaryotic 60 s acidic ribosomal proteins are called ribosomal P proteins because they can be phosphorylated [41]. P protein is a structural constituent of the ribosome and is involved in nucleic acid binding and translation. Furthermore, the presence of P proteins has been reported to be related to infections caused by protozoa since anti-P-antibodies were detected against *Trypanosoma cruzi* and *Leishmania* species [42, 43].

Categorised under metabolic process and catalytic activity, ES proteins of *E. histolytica* with proteolytic properties were also identified. Among them, we have identified a protein family called cysteine proteinase (EHI_168240, and EHI_074180). The roles of cysteine proteinase include the degradation of fibrinogen, collagen, and the basement membrane matrix [10, 12, 13]. When proteinase or proteases of parasites are expressed on the cell surfaces and/or released into the extracellular environments, they often damage the host. An example is the action of *E. histolytica* cysteine proteinase on the disruption of the cysteine-rich MUC2 polymer of the host tissue [44].

Interestingly, we have detected an F-actin capping protein beta subunit (EHI_005020) that is involved in a variety of biological functions including metabolic process, biological regulation, cellular component organization/biogenesis, cellular process, and developmental process. In addition, it is also involved in binding activity by molecular function. The capping protein is involved in the motility of *E. histolytica* and is known to inhibit the elongation of the actin filament for the motility of the parasite [45]. The capping proteins are also part of a larger protein complex called Wiskott–Aldrich Scar Homology (WASH) whereby the complex is associated with the endosomes [46], thus explaining the presence of the capping protein in the extracellular environment.

We have also detected Calmodulin (EHI_023500) in the ES proteins of *E. histolytica*. Calmodulin (CaM) is involved in many categories under biological and molecular functions, including metabolic process, cellular process and localization, catalytic activity, and binding. CaM is associated with the secretion of electron-dense granules containing collagenase, as well as the growth and encystation of *Entamoeba* spp. [47, 48]. Makioka et al. [49] demonstrated the function of CaM in the excystation and metacystic development of *E. invadens* as a model for *E. histolytica.*

Within the biological function category of proteins related to response to stimulus, several heat shock proteins that respond to stress were identified. The 70 kDa heat shock protein (EHI_199590), also known as BiP, has been suggested to be involved in encystation in an experiment using *E. invadens* which showed 88% sequence homology to *E. histolytica* [50]. In addition, heat shock protein (HSP) 70 of *E. histolytica* was reported to stimulate immune responses in patients with amoebiasis [51]. Furthermore, amoebic HSPs have been suggested to be involved in the degradation of cytoskeletal proteins for the purpose of encystation [52, 53]. On the other hand, the presence of HSP may not indicate stress response, as a previous study demonstrated that HSP was present in both heated and unheated cells of *Amoeba proteus* [54].

By molecular functional analysis, a notable number of proteins that were classified under the binding category were actin binding proteins. Previously, Váazquez et al. [55] described four actin binding proteins (vinculin, α-actinin, tropomyosin and myosin I) of amoebic adhesion plaques/plates. In the present study, we identified actin-binding proteins (EHI_168340), cofilin/tropomyosin family (EHI_186840), and myosin heavy chain (EHI_110180). These proteins do not only exist on the surface of *E. histolytica* as a component in adhesion plaques, but also aid the parasite in locomotion and invasion of the host tissue [55].

**Fig. 5:**
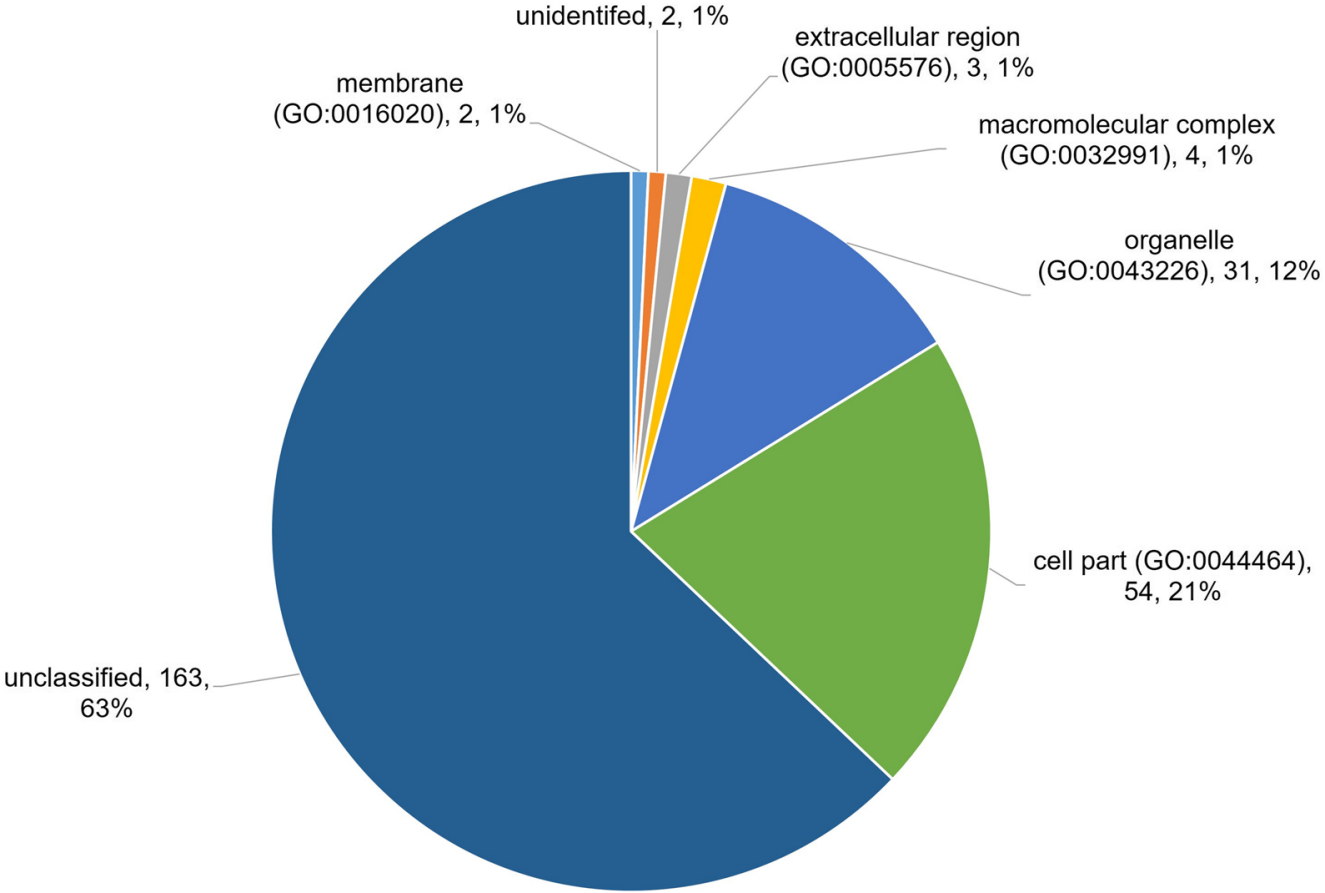
Cellular component. Classification of ES proteins based on their association with cellular components. The classifications were generated using Panther version 11.0 released 2016-07-15

Analysis by cellular component (Fig. 5) characterizes proteins according to their subcellular location and macromolecule complex level. A large number of proteins detected (54 proteins) belonged to the category of ‘cell part’, which includes intracellular and plasma membranes, followed by 31 proteins that are associated with the organelle (cytoskeleton), 4 extracellular region proteins, 2 proteins that are related to the membrane, and 3 proteins that are affiliated with the macromolecular complex.

This study has revealed proteins considered as surface membrane proteins. Slightly more than half of the proteins that we identified were also *E. histolytica* membrane surface proteome reported by Biller [39], such as disulphide isomerase, enolase, heat shock proteins, malate dehydrogenase, triosephosphate isomerase, thioredoxin, and superoxide dismutase. The identification of membrane-related proteins in the ES products may be due to membrane recycling. An example in *Acanthamoeba* demonstrated by Hohman and Bowers [56] showed that proteins secreted when trapped in the shuttle vesicles were transported from secondary lysosomes to the surface membrane.

## Conclusion

The ES proteome of *E. histolytica* was studied using two methods of mass spectrometry analyses. The results showed that ES proteins are involved in the colonisation and evasion of the host immune system as well as in encystation and excystation. The results of this study can further our understanding of the pathogenesis of *E. histolytica*. In future research, ES proteins from parasites isolated from an infected animal can be analysed in order to provide deeper insights into the host-parasite interactions. Furthermore, real patient samples could be analysed to detect the presence of some of the identified and unknown proteins found in this study.

## Additional files

**Additional file 1.** Protein score from LC-MALDI-TOF/TOF.

**Additional file 2.** Protein score from LC-ESI-MS/MS.

**Additional file 3.** Peptide summaries from MALDI and ESI.

**Additional file 4.** De novo peptides from ESI.

**Additional file 5.** Contaminant proteins analysis.
